# Supplementary material for: Persistent CO2 emissions and hydrothermal unrest following the 2015 earthquake in Nepal
Source: Nat Commun. 2018 Jul 27;9:2956. doi: 10.1038/s41467-018-05138-z (PMC6063904; doi:10.1038/s41467-018-05138-z)
Supplement: Supplementary file 1 — Supplementary Information [file 41467_2018_5138_MOESM1_ESM.pdf]

SUPPLEMENTARY INFORMATION

Persistent CO_2 emissions and hydrothermal unrest following the 2015 earthquake in Nepal

Girault et al.

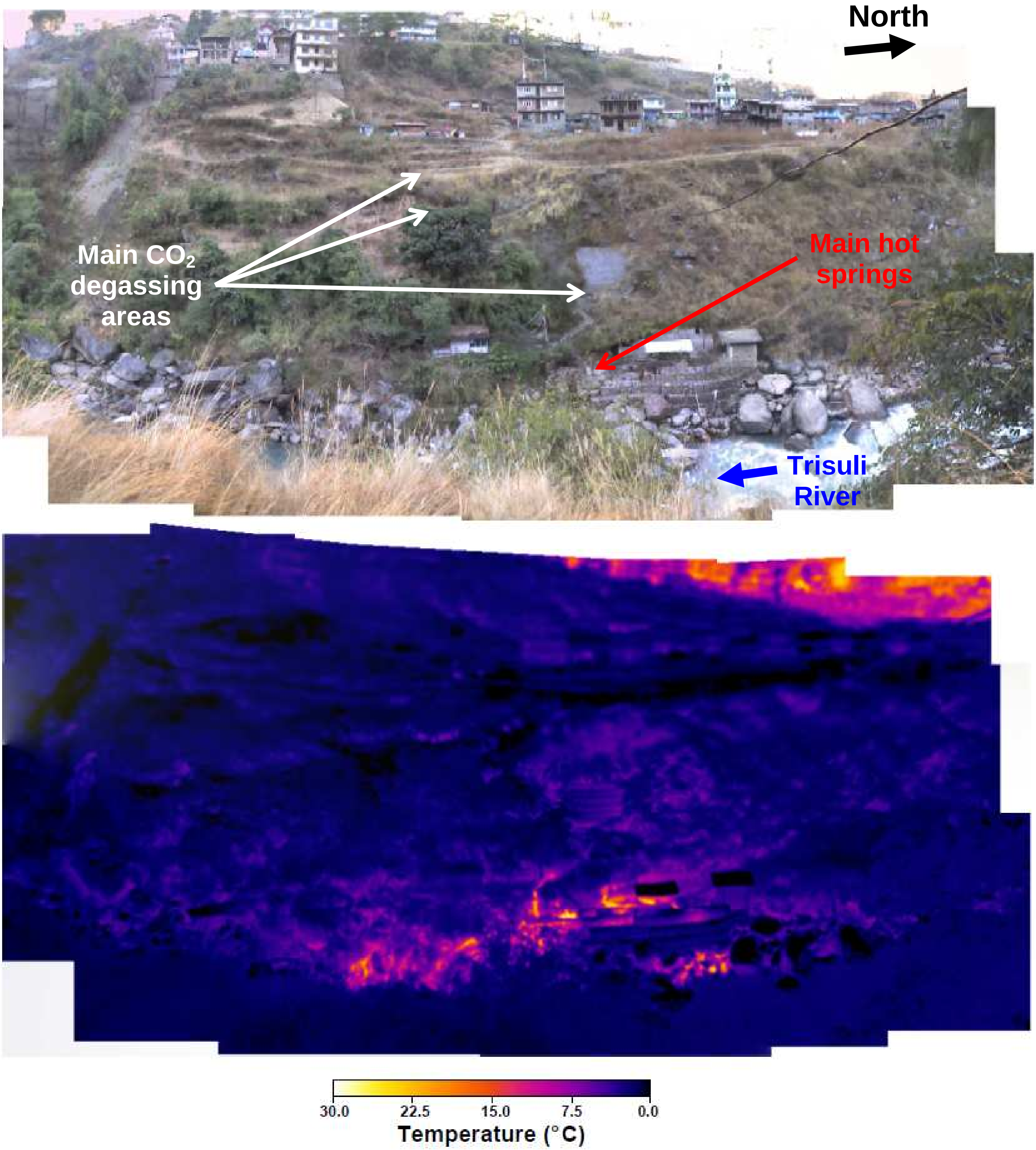

Supplementary Figure 1 | Optical and thermal infrared images of the main hot springs and of the diffuse degassing structures (GZ1-2) on the alluvial terrace in Syabru-Bensi, Central Nepal. Spots of higher surface temperature compared with the ambient air temperature (5°C at the time of the picture, January 2016) are visible. CO_2 emission on this terrace is shown in Fig. 2.

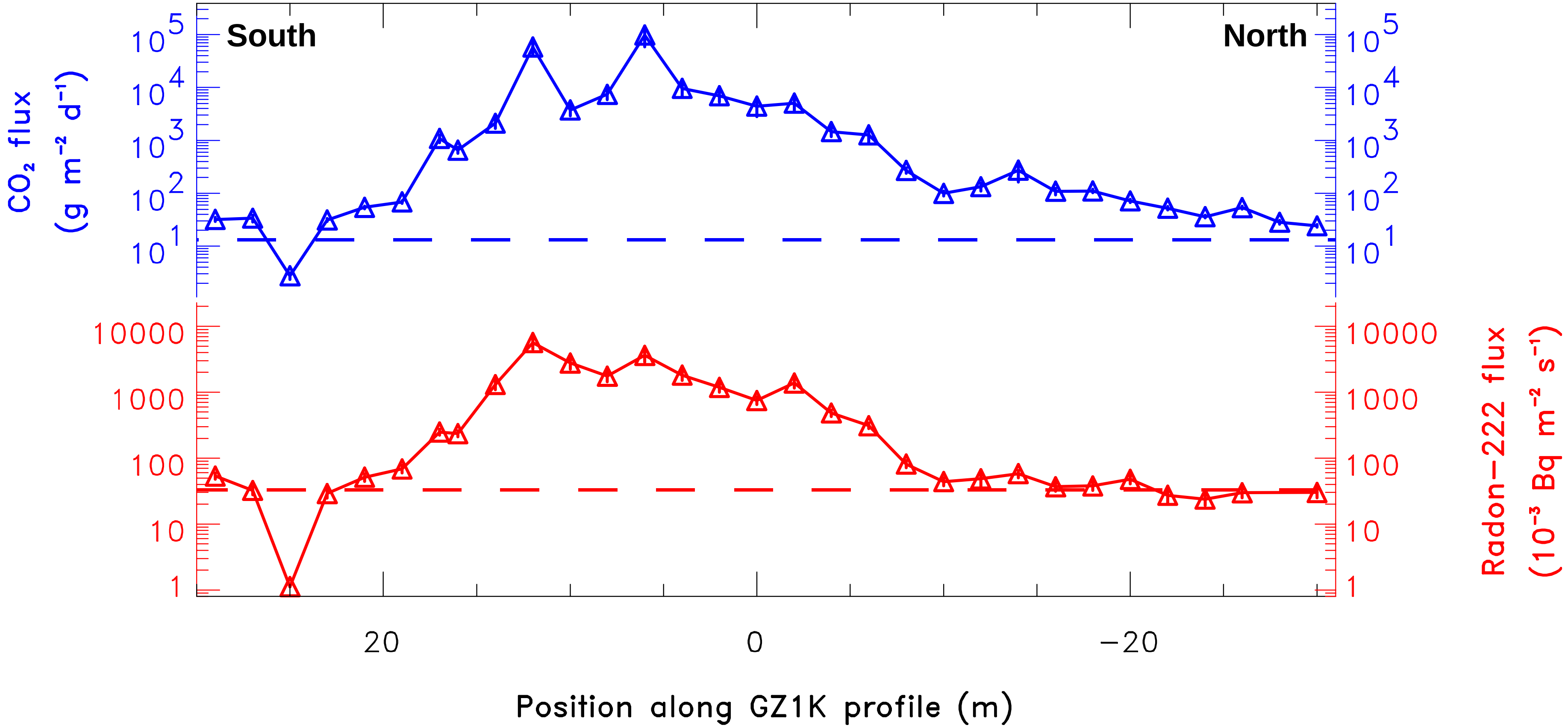

Supplementary Figure 2 | Carbon dioxide and radon-222 fluxes along GZ1K profile on the alluvial terrace in Syabru-Bensi, Central Nepal. The highest CO_2 and radon fluxes correspond to measurement points K+6 (position +6 metres) and K+12 (position +12 metres), as detailed in the main text (see also Figs. 2 and 3). Dashed lines represent CO_2 and radon flux continental averages[34] of 10 g m^{-2} d^{-1} and 22×10^{-3} Bq m^{-2} s^{-1}, respectively.

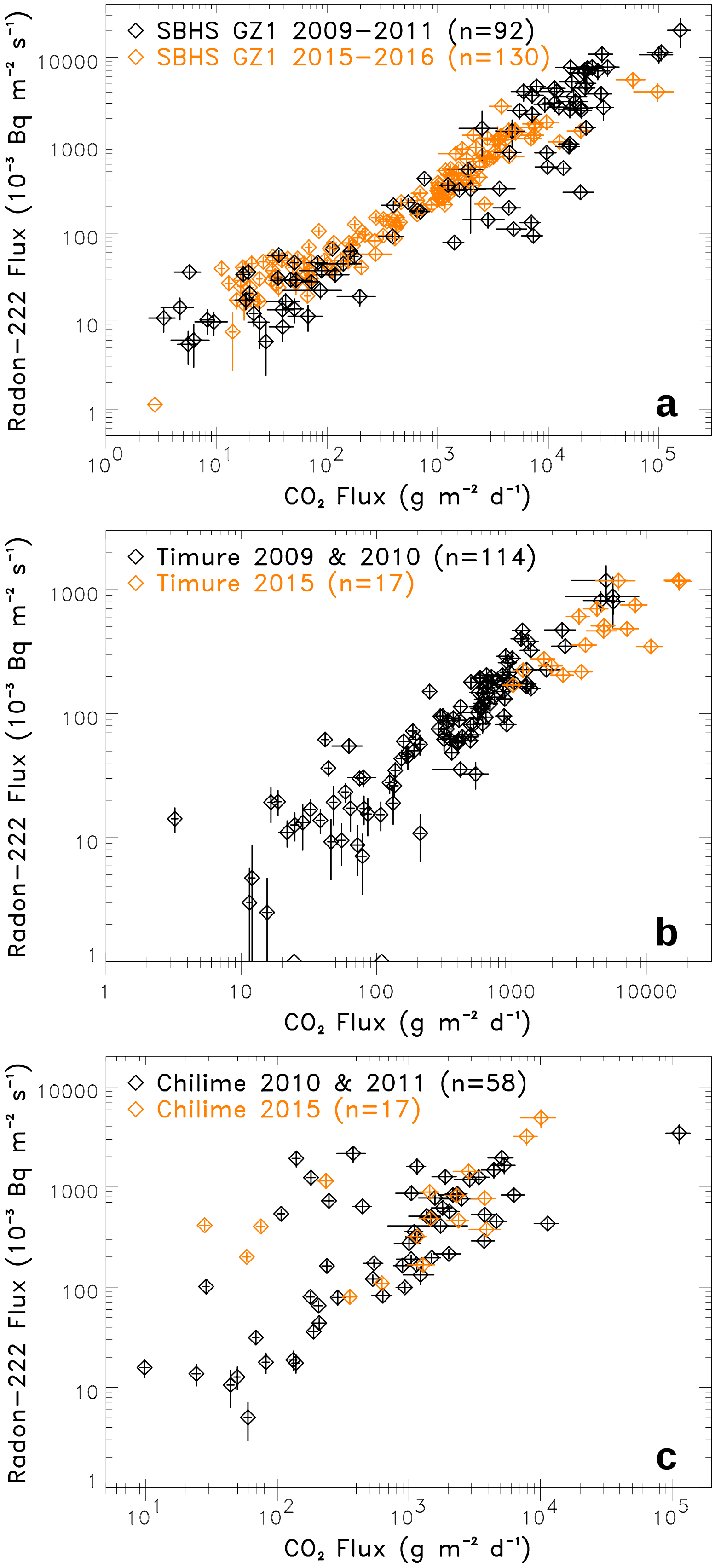

Supplementary Figure 3 | Carbon dioxide flux versus radon-222 flux, before and after the Gorkha earthquake, for (a) the alluvial terrace in Syabru-Bensi, (b) the northern profile in Timure and (c) the Chilime site, Central Nepal. In Syabru-Bensi where the number of data is sufficiently large, CO_2 and radon fluxes are similarly correlated before and after the earthquake, suggesting similar gas transport mechanisms, source and travel time[37].

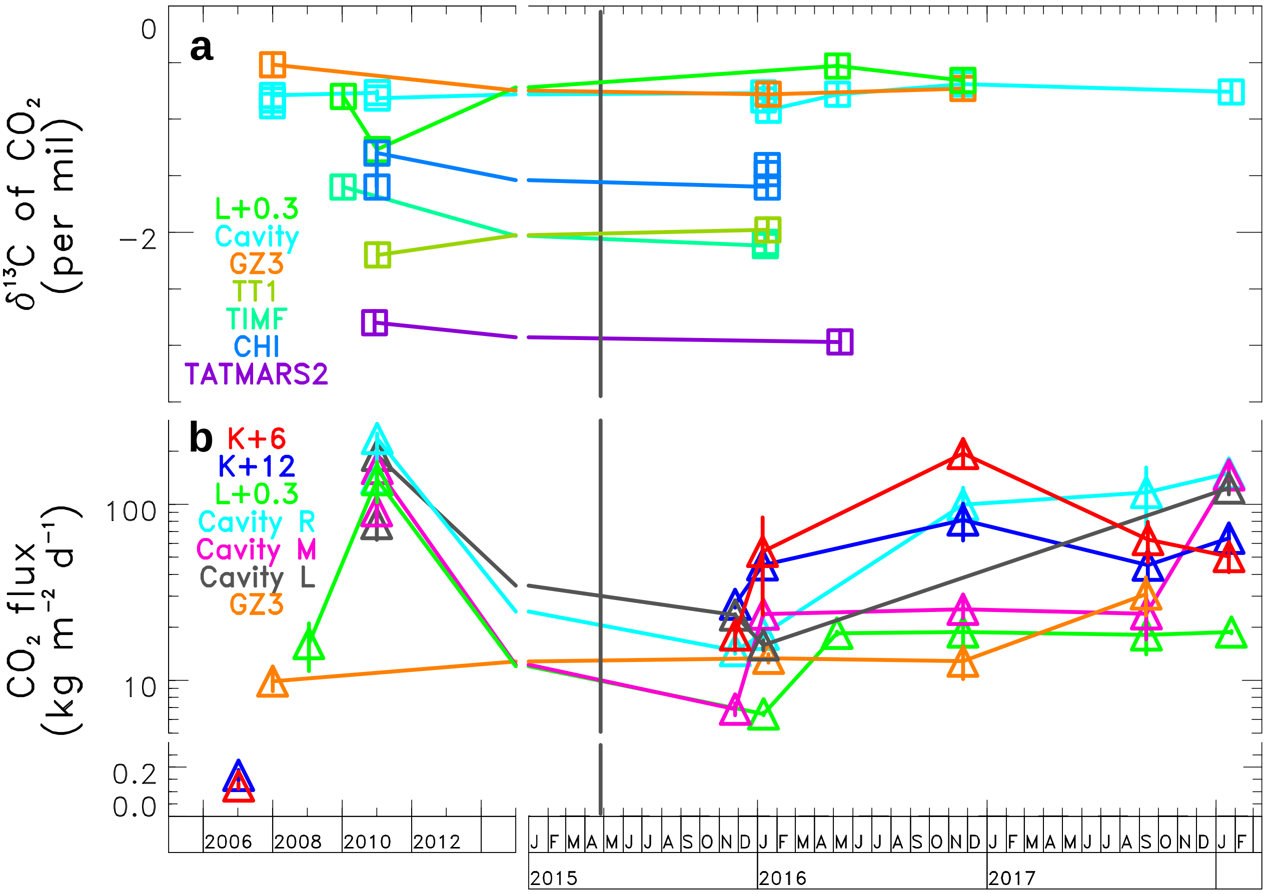

Supplementary Figure 4 | Available time-series of carbon dioxide fluxes and carbon isotopic ratios at selected gas emission sites in Central Nepal. Values of $\delta^{13}C$, relative to V-PDB, of the gaseous CO_2 and surface CO_2 fluxes from 2006 to 2018 are shown in (a) and (b), respectively. Names and locations of CO_2 emission sites are given in Table 1 and Supplementary Table 1: Syabru-Bensi GZ1 (Cavity, Cavity R, M and L, K+6 and K+12), GZ2 (L+0.3) and GZ3 (GZ3); North Syabru (TT1); Timure (TIMF); Chilime (CHI); and Bahundanda (TATMARS2). To first order, the $\delta^{13}C$ values remain relatively similar at sites showing strong post-seismic effects (Syabru-Bensi, Timure and Chilime in the Upper Trisuli valley), and at sites where no post-seismic changes were observed (Bahundanda in the Marsyandi valley). Increases of $\delta^{13}C$ values of 17±2% in Syabru-Bensi and of 30±8% in Bahundanda are, however, observed after the earthquake. In Syabru-Bensi, after the earthquake, the two CO_2 fluxes (K+6 and K+12) located on the alluvial terrace above increased (see also Fig. 3), while the three CO_2 fluxes located in the cavity below decreased significantly by a factor of 13±3 on average. The values of CO_2 flux in the cavity returned to values measured before the earthquake in January 2018, more than 2.7 years after the mainshock. On the terrace above, the values of CO_2 flux in January 2018 remained greater that the pre-earthquake values.

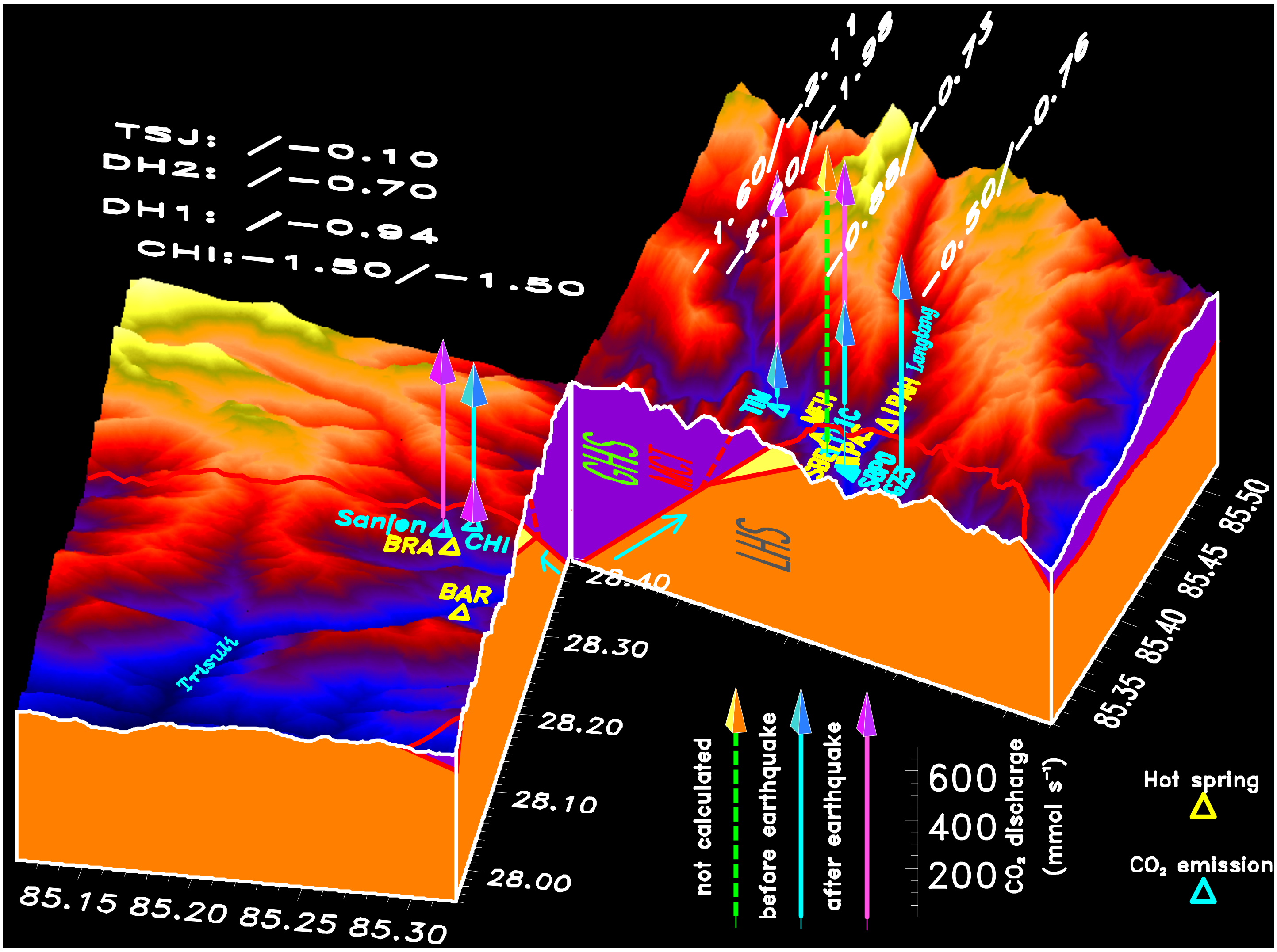

Supplementary Figure 5 | Overview of carbon dioxide emissions in the Upper Trisuli valley, Central Nepal. The bloc diagram corresponds to the top right inset of Fig. 1 and shows total CO_2 discharges, estimated before and after the Gorkha earthquake, in Syabru-Bensi, Timure, Chilime and Sanjen. Numbers refer to $\delta^{13}C$ of CO_2, relative to V-PDB, before/after the earthquake. The Main Central Thrust (MCT) and the Ramgarh Thrust are shown in red. Rocks of the Lesser Himalayan Sequence (LHS) and of the Greater Himalayan Sequence (GHS) are shown in the bloc edges in orange and in purple, respectively[29]. The oldest rocks of the LHS, Paleoproterozoic to Early Proterozoic augen gneiss, are displayed in yellow. Topography is smoothed from the Shuttle Radar Topography Mission (SRTM) Digital Elevation Model (DEM). Details of the main CO_2 emission sites and of the hot spring sites are given in Tables 1 and 2 and in Supplementary Tables 1 and 2, respectively. The $\delta^{13}C$ data remain similar after the earthquake (see also Supplementary Fig. 4), with the exception of $\delta^{13}C$ increases in Syabru-Bensi (17±2%), as well as in Bahundanda (30±8%), suggesting less fractioning and/or faster gas transport. Figure performed using PV-WAVE® software (Rogue Wave).

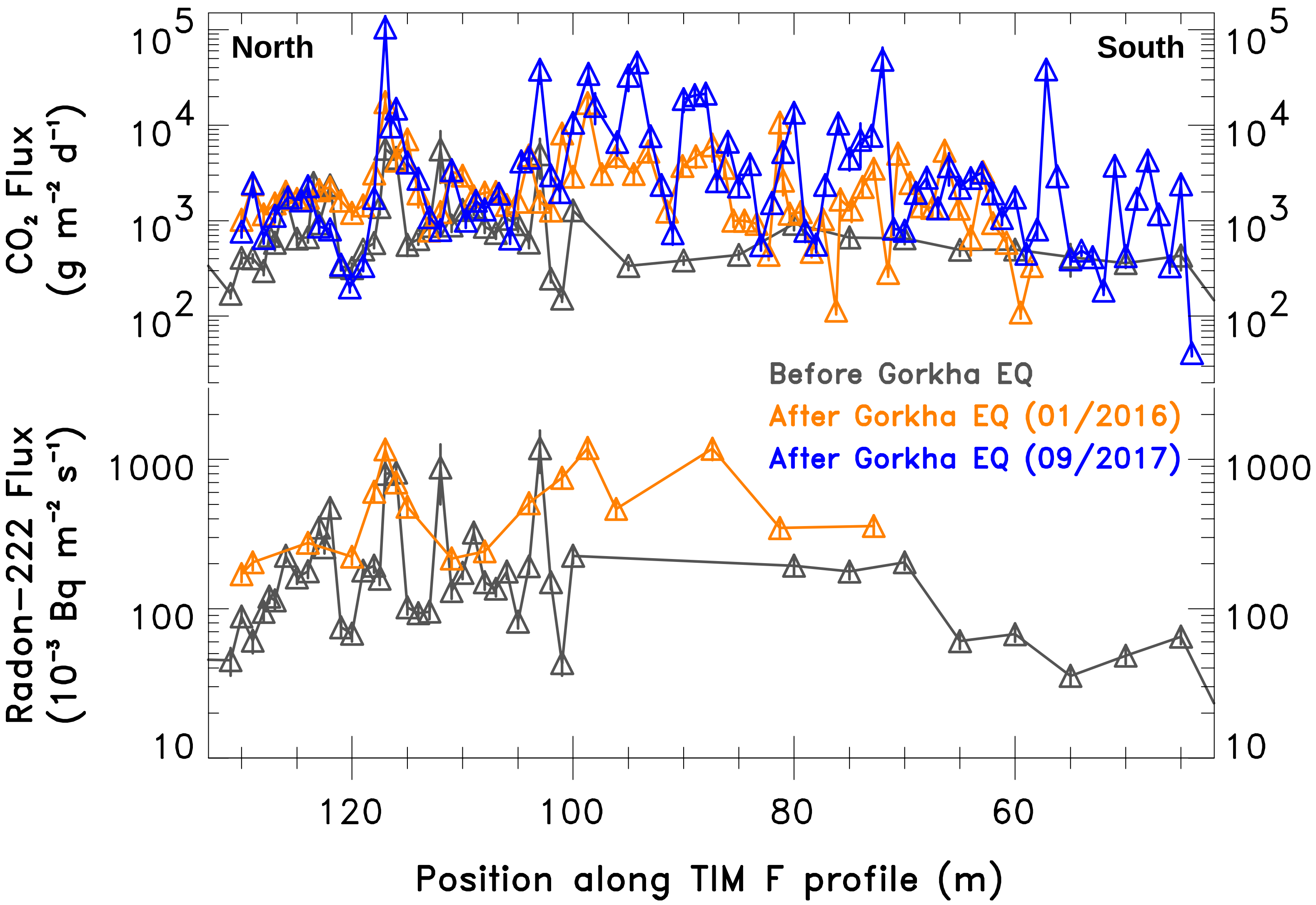

Supplementary Figure 6 | Carbon dioxide and radon fluxes along the northern profile in Timure, before and after the Gorkha earthquake, Central Nepal. Along the profile, described elsewhere[51], the CO_2 emissions are significantly larger after the earthquake by a factor of 3±1 in January 2016, and by a factor of 8±3 in September 2017.

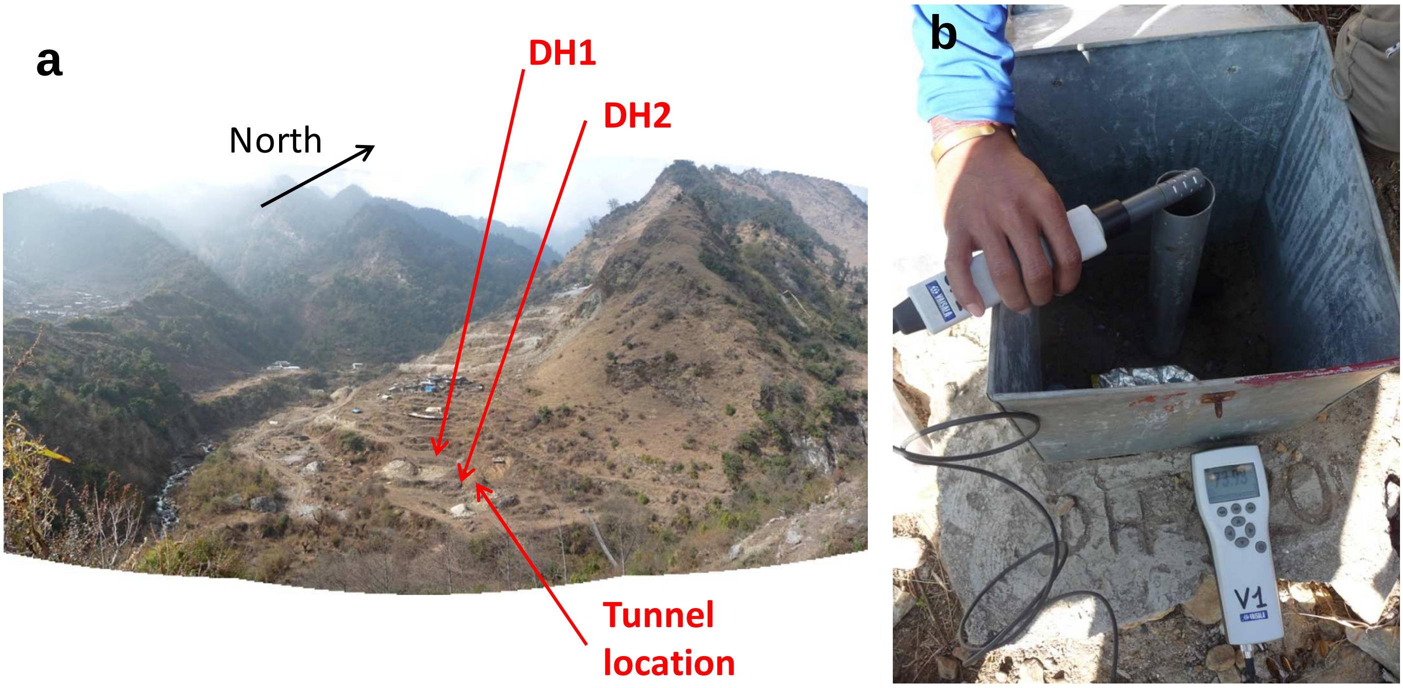

Supplementary Figure 7 | The Sanjen Hydroelectric Project site, Central Nepal, showing post-seismic CO_2 outbursts from two piezometers and in a tunnel. (a) General location of the site with the two piezometers and the tunnel (being excavated), where large CO_2 emission was detected. (b) Picture illustrating the large CO_2 concentration (>98%) at the top of DH1 piezometer. Outburst of CO_2 from DH1-2 piezometers started between the 1st and the 8th of November 2015.

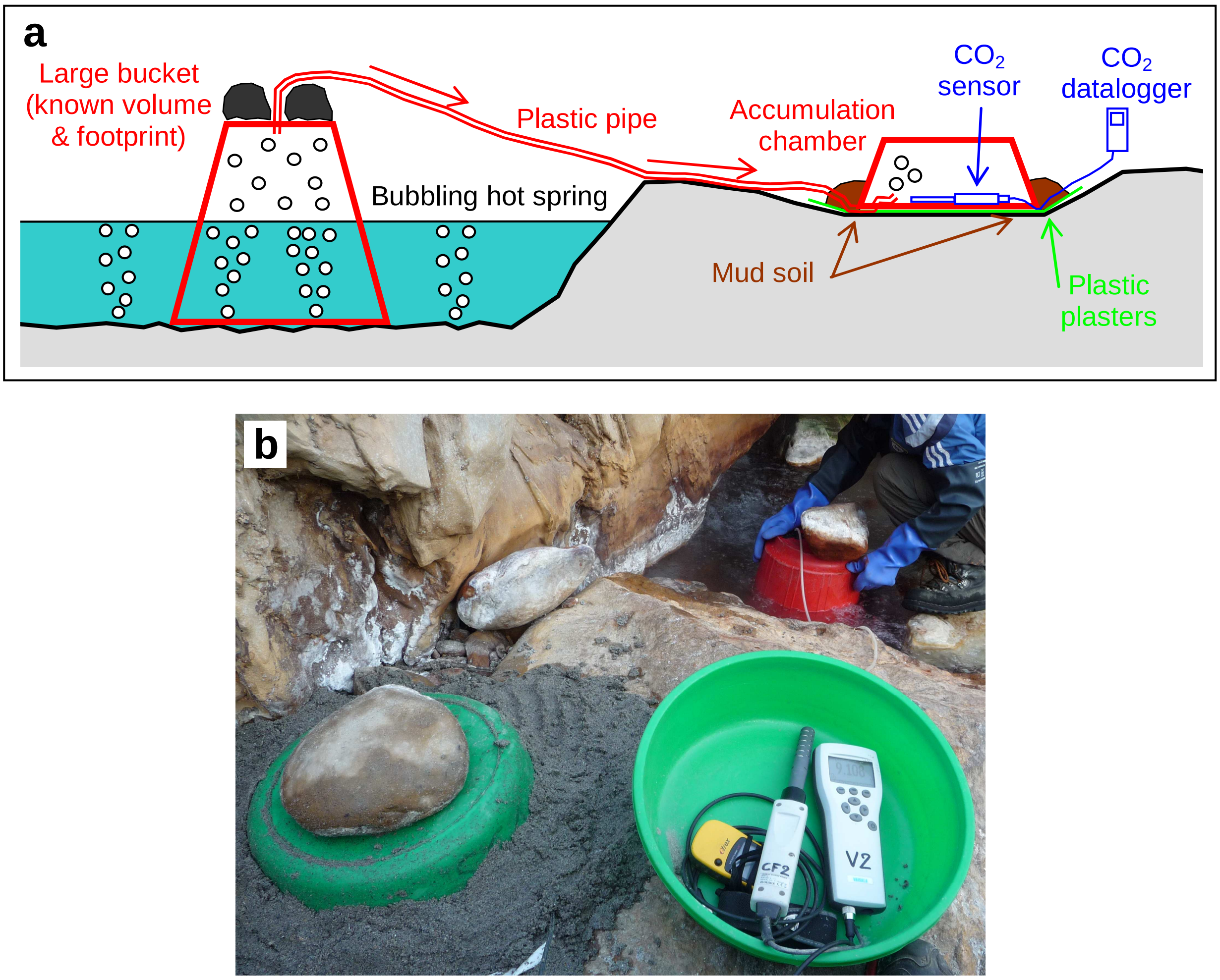

Supplementary Figure 8 | Experimental protocol for the measurement of carbon dioxide flux through a water layer. (a) Sketch of the measurement method using a large bucket connected with a pipe to the accumulation chamber. (b) Example of CO_2 flux measurement through a water layer carried out in Machhakhola (Budhi Gandaki valley) in January 2018.

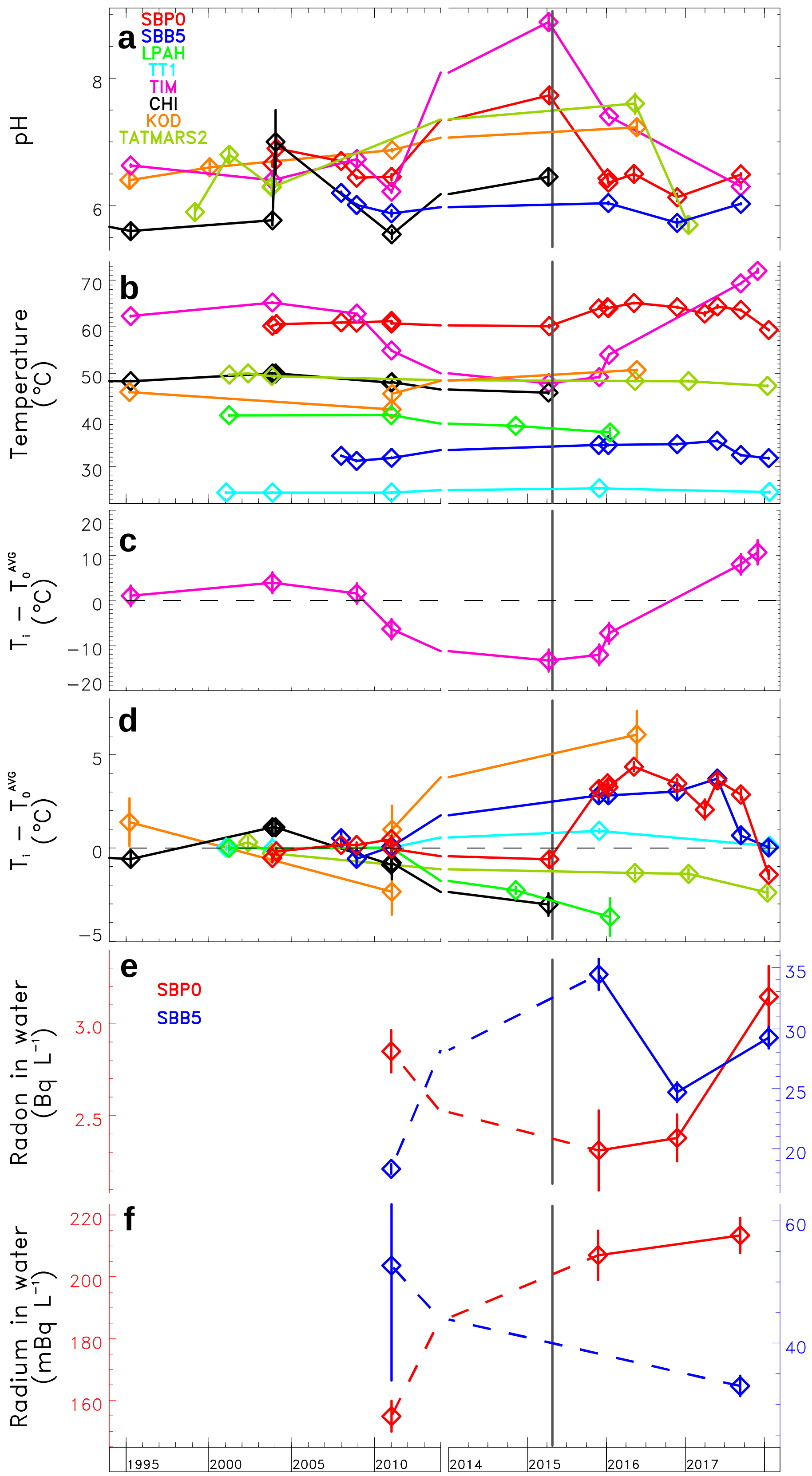

Supplementary Figure 9 | Available time-series of pH, temperature and dissolved radon-222 and radium-226 concentrations in water for hot springs of Central Nepal. Water pH and temperature are shown in (a) and (b), respectively. Temperature difference with the mean temperature value before the earthquake is plotted for (c) Timure and (d) the seven other springs. Dissolved radon-222 and radium-226 concentrations in water are shown in (e) and (f), respectively, separately for SBP0 and SBB5 Syabru-Bensi hot springs. The vertical grey line indicates the Gorkha earthquake. Spring names and locations are given in Table 2 and Supplementary Table 2. Temperature time-series of the Chilime hot spring is the longest and starts[64] on March, 1st 1980 with similar temperature value of 49.0±0.2°C, while the spring was first studied by Le Fort[63] on October, 1st 1975. Water-cooling was observed a few weeks before the earthquake at four hot springs of the Upper Trisuli valley: Syabru-Bensi (SBP0), Timure (TIM), Langtang (LPAH) and Chilime (CHI).

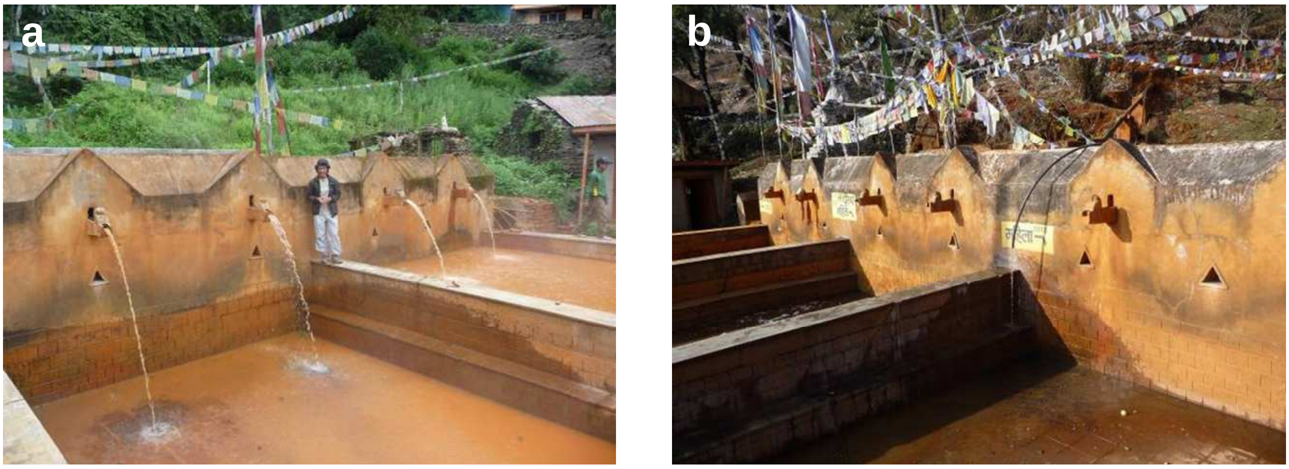

Supplementary Figure 10 | The Chilime hot spring, Central Nepal, stopped flowing after the Gorkha earthquake, between the 10th and the 20th of October 2015. (a) Picture showing the pipes, the cemented basins and the spring flow rate of about 5 L s^{-1} with mean temperature of 48.9±0.4°C before the earthquake (picture taken in August 2010). (b) Situation after October 2015, continuing in January 2018 (picture taken in January 2016).

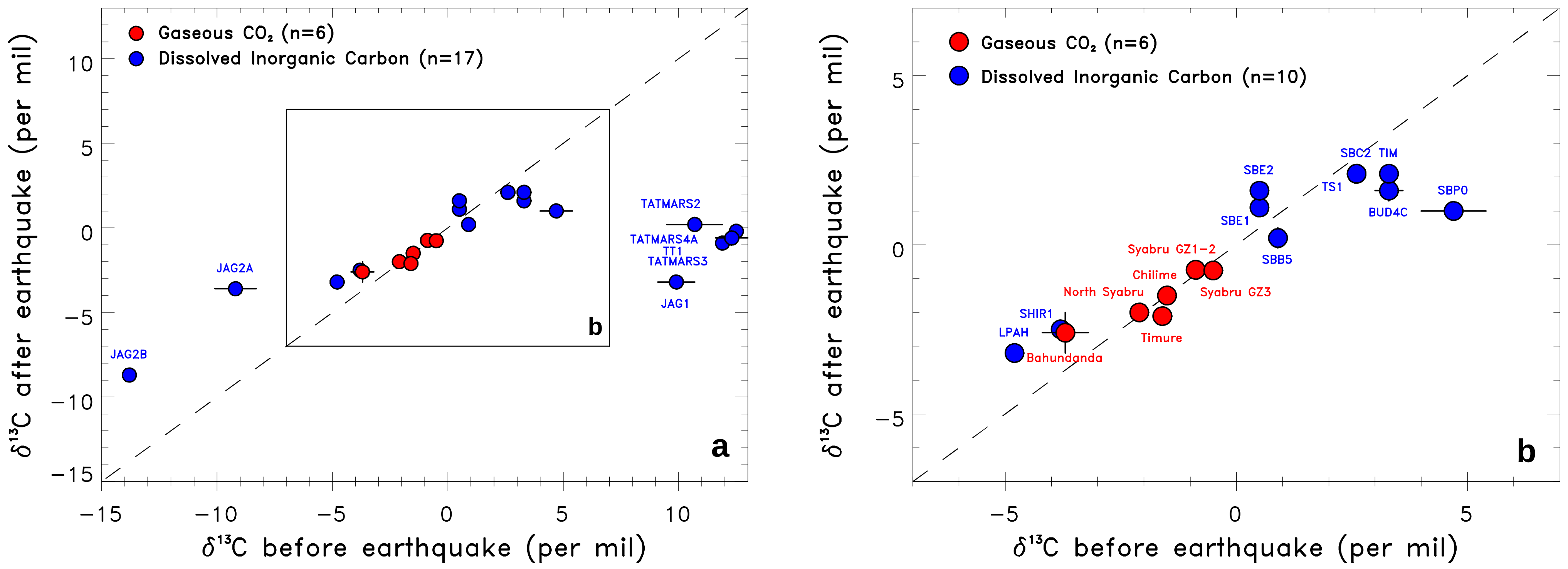

Supplementary Figure 11 | Comparison of carbon isotope ratios in the gas and water phases in Central Nepal before and after the Gorkha earthquake. (a) $\delta^{13}C_{gas}$ (red) and $\delta^{13}C_{DIC}$ (blue) of our whole data-set. (b) Enlargement of the central part of (a). Data are summarised in Tables 1 and 2 and in Supplementary Tables 1 and 2.

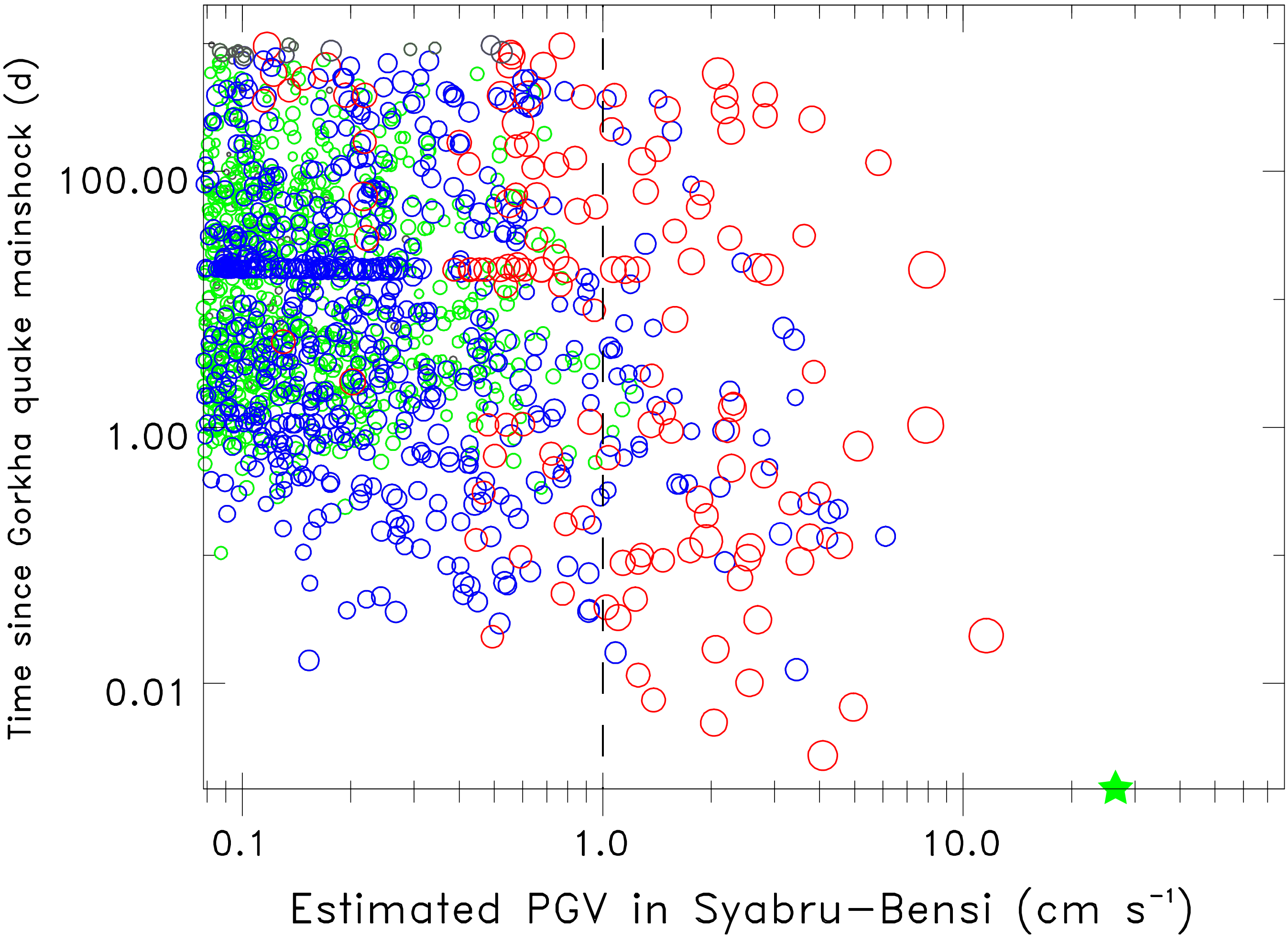

Supplementary Figure 12 | Estimated vertical Peak Ground Velocity in Syabru-Bensi for the seismic events during the first 31 months following the Gorkha earthquake. The time since the mainshock is expressed in days in logarithmic scale. The star corresponds to the Gorkha earthquake (PGV of 26.5 cm s^{-1}; Supplementary Table 3). Red, blue and green circles correspond to aftershocks with local magnitudes M_L>5, 4<M_L≤5 and 3<M_L≤4, respectively. We used the aftershock catalogue of the first 31 months after the mainshock generated by the NSC seismic network (from April 25, 2015 to December 31, 2017). PGV is estimated based on equations given in ref. 58 (Methods).

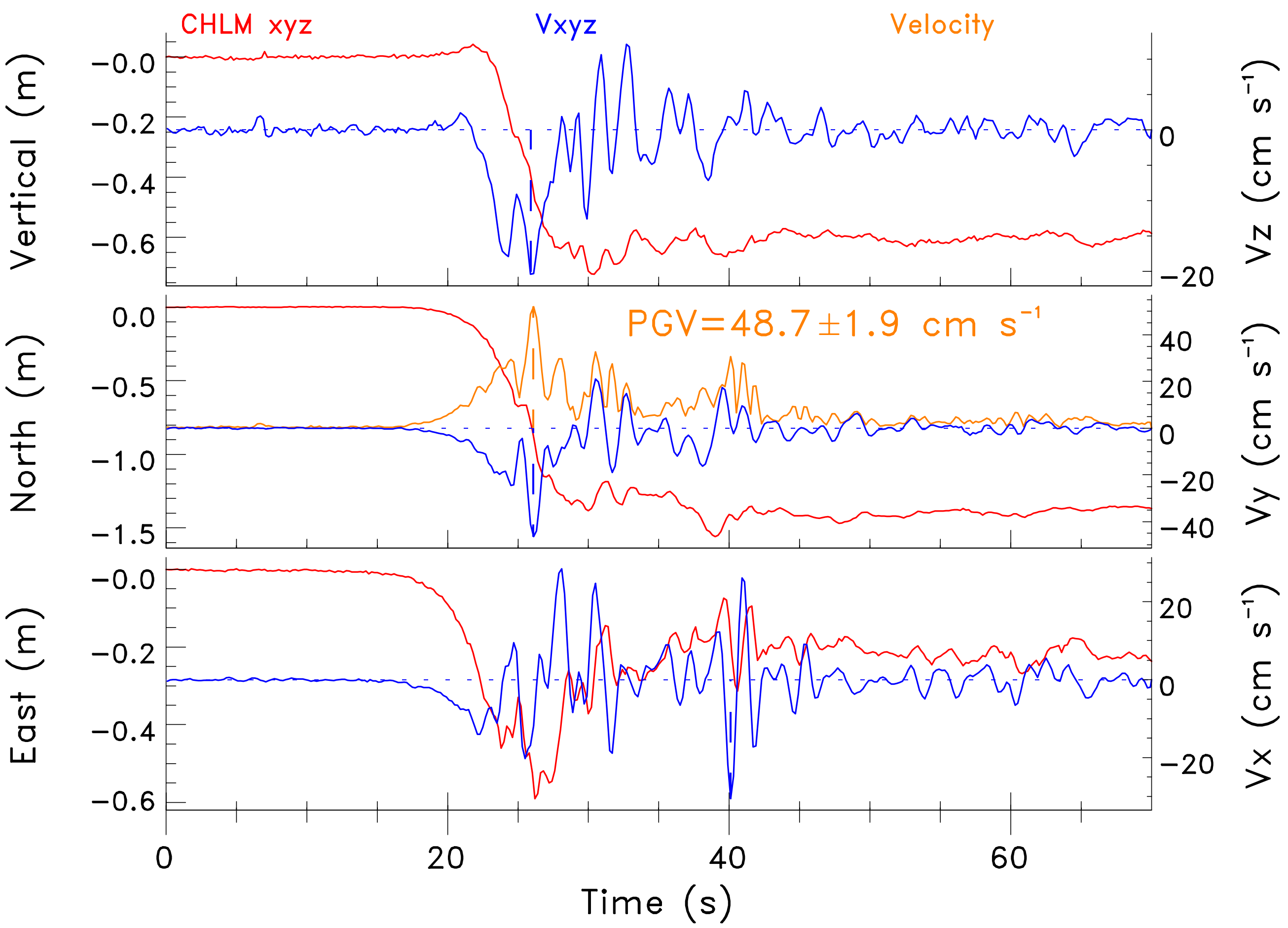

Supplementary Figure 13 | Estimated vertical Peak Ground Velocity based on the GPS data of CHLM station near Chilime. A large PGV of 49±2 cm s^{-1} is obtained at CHLM station using the method of ref. 45.

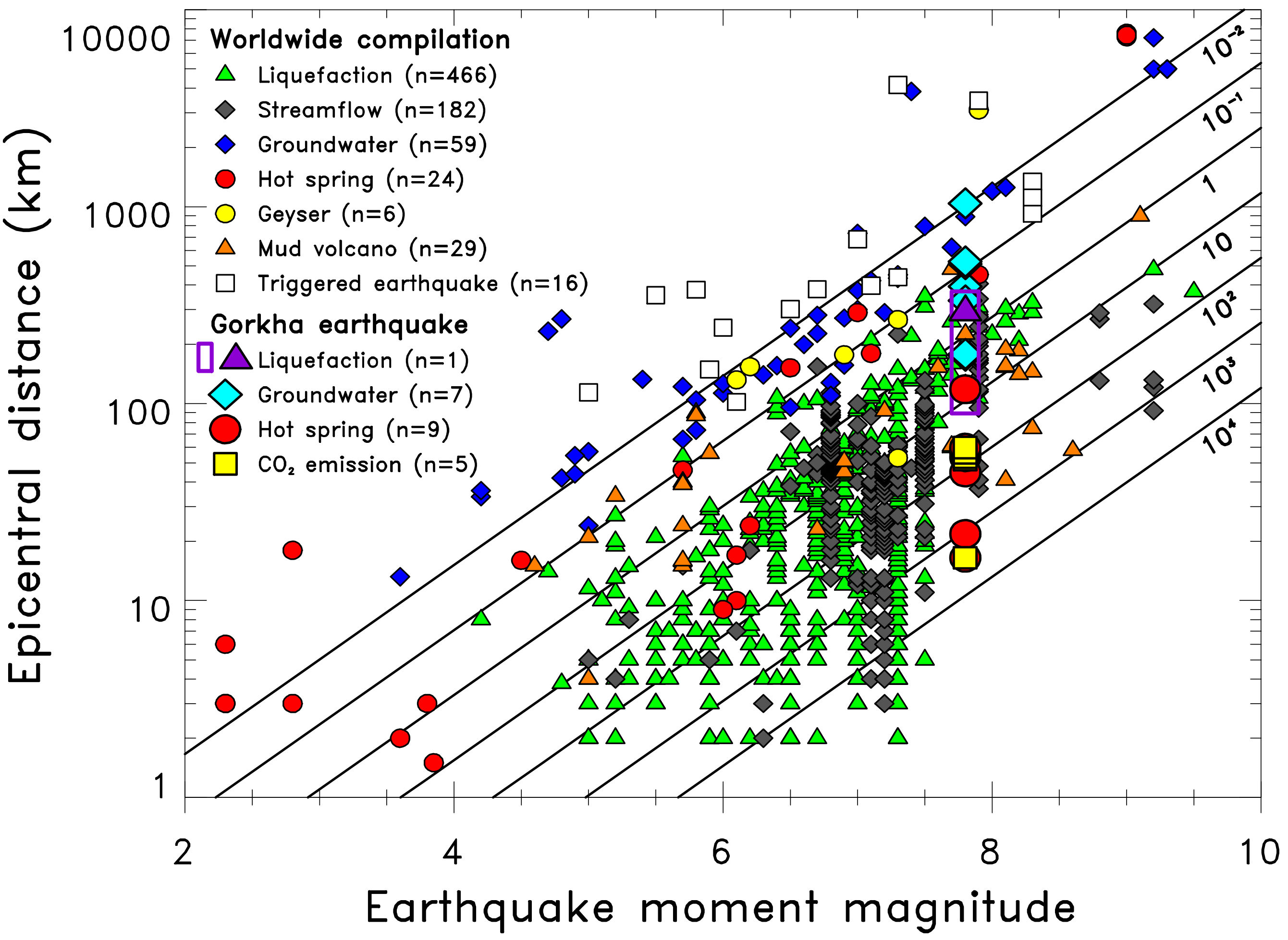

Supplementary Figure 14 | Gorkha earthquake-induced responses plotted on earthquake magnitude versus distance. We inserted the worldwide earthquake-triggered hydrological changes compiled[10,19,22,56]. Reported liquefaction (n=1) and groundwater (n=7) responses to Gorkha earthquake[40] are also plotted. Lines show contours of SED expressed in J m^{-3} and calculated from ref. 56 (Methods). Our observations of significant changes in the CO_2 emissions at five locations are the first reported earthquake-induced gaseous changes in a non-volcanic region.

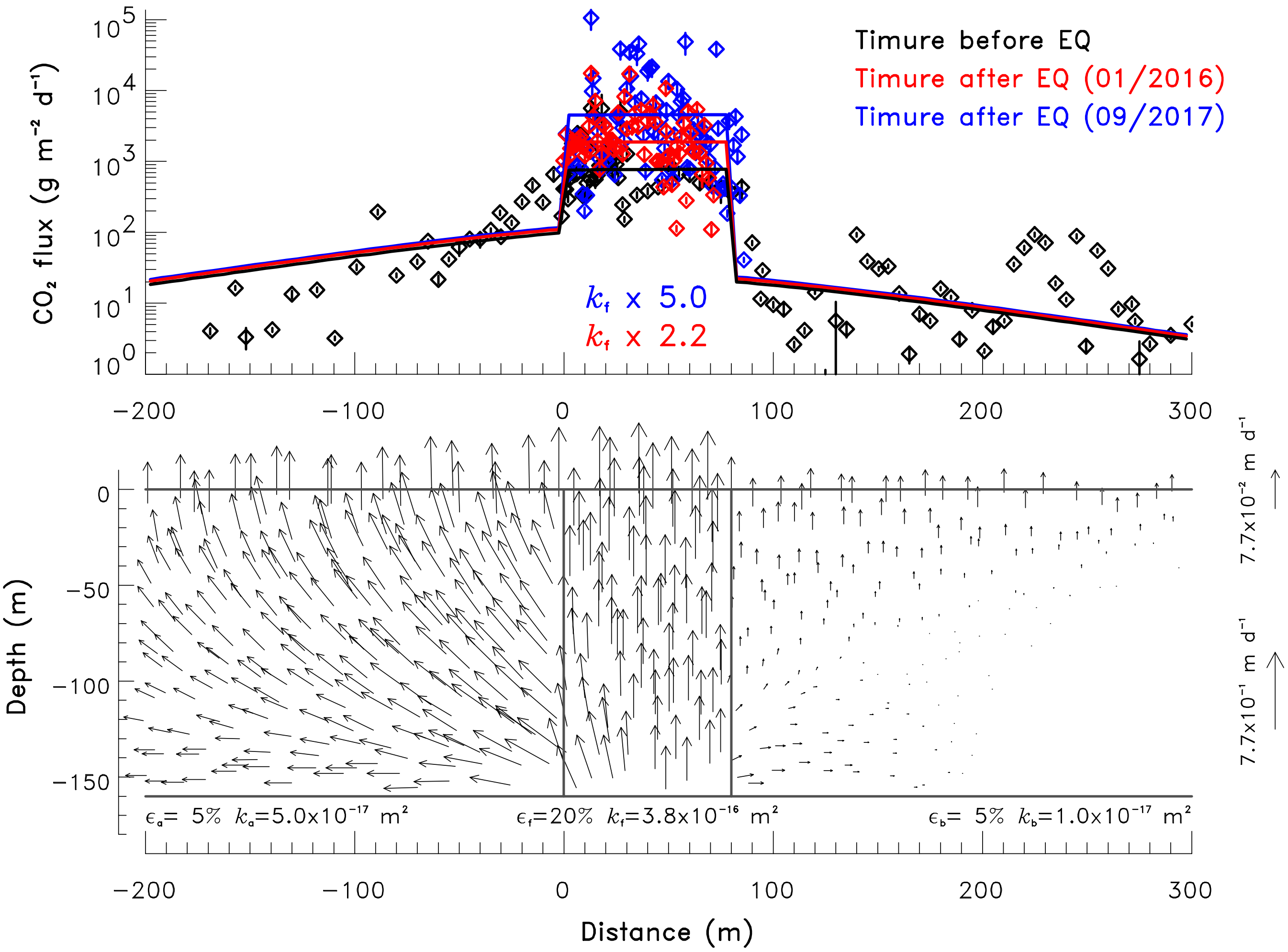

Supplementary Figure 15 | Effect of permeability change on the surface carbon dioxide fluxes in Timure before and after the Gorkha earthquake. We calculated the advective gaseous CO_2 transport using the simplified 2-D fault model described in ref. 37. (bottom) Fluid velocity versus depth, expressed in metre per day; (top) CO_2 fluxes on the ground as a function of horizontal distance. Mean CO_2 fluxes measured along the northern profile of Timure before[30,51] and after the earthquake are plotted (diamond). An increase of fault permeability, k_f, by a factor of 2.2 after the earthquake accounts for the three times larger CO_2 fluxes in the centre of the profile obtained in January 2016. Similarly, an increase of fault permeability by a factor of 5.0 after the earthquake accounts for the eight times larger CO_2 fluxes in the centre of the profile obtained in September 2017.

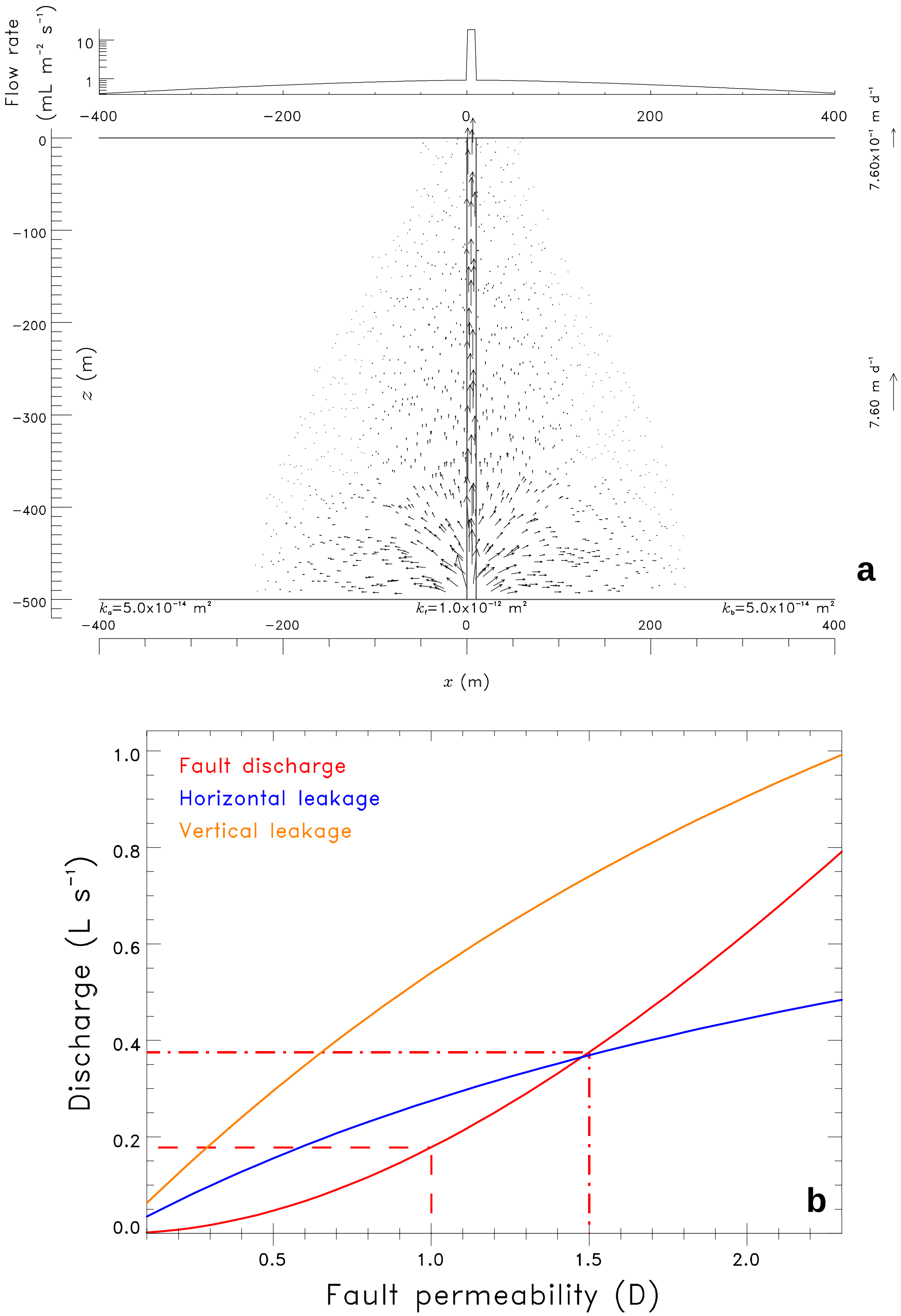

Supplementary Figure 16 | Effect of permeability change on spring water flow rate at the surface. We adapted the 2-D fault model briefly recalled in Methods and described elsewhere[37] to the calculation of water flow rate. (a) Fluid velocity versus depth, expressed in metre per day (bottom); surface water flow rate as a function of horizontal distance (top). (b) Calculated surface water discharge for varying fault permeability. Fault permeability is expressed in Darcy. A 10% increase of fault permeability is sufficient to increase water flow rate by 20%, as observed for the main Syabru-Bensi hot spring (SBP0). If permeability of the surrounding media decreases, the flow rate increases, suggesting that the new springs in Syabru-Bensi may be neighbouring leakages of the main conduit.

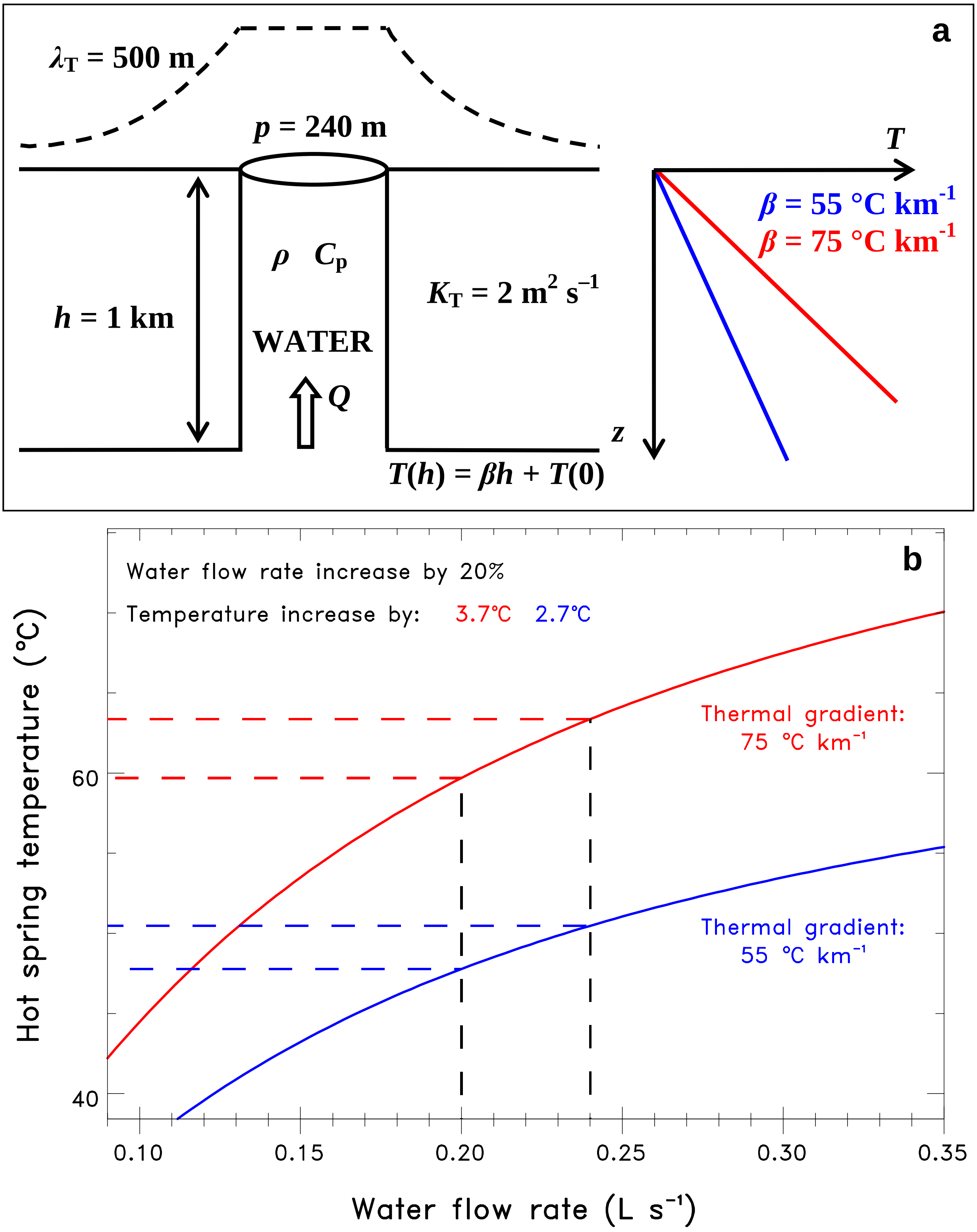

Supplementary Figure 17 | Effect of water flow rate change on hot spring temperature. (a) Sketch of the first-order model described in Methods. (b) Calculated hot spring temperature as a function of water flow rate for two thermal gradients. Using a thermal gradient of 75 °C km^{-1} (ref. 61), a 20% increase of water flow rate increases water temperature by 3.7°C, similar to what is observed for the SBP0 hot spring in Syabru-Bensi, as shown in Fig. 3 and Supplementary Fig. 8. A temperature increase of 2.7°C, similar to what is observed for the SBB5 hot spring in Syabru-Bensi, can be obtained with a smaller thermal gradient of 55 °C km^{-1}.

Supplementary Table 1 | Available data of CO_2 flux, total CO_2 discharge, CO_2 concentration and carbon isotopic ratio of CO_2 emissions before and after the Gorkha earthquake at nine hydrothermal sites in Central Nepal.

Site	Location	Coordinates	Before/After Gorkha Earthquake	CO_2 Flux ($g\ m^{-2}\ d^{-1}$)			CO_2 Discharge (10^{-3} mol s^{-1})	CO_2 Concentration (%)	$\delta^{13}C$ of CO_2 (V-PDB) (‰)			Post-seismic Effect
				N_{meas} (n_{point})	Min/Max Range	Geometric Mean			N_{meas} (n_{point})	Min/Max Range	Geometric Mean	
**Marsyandi Valley**												
Khudi	Probi Hot Springs Terrace	28.34547°,84.33112°,1250m	After (01/2018)	10(8)	110/7020	680±40	>60±15	38±1	1(1)		–2.6±0.1	Unknown
	Probi Hot Spring PRO2	28.34515°,84.33135°,1250m	After (01/2017)	0				64±1	1(1)		–3.7±0.1	Unknown
Bahundanda	Main Hot Springs Terrace	28.33992°,84.39775°, 930m	Before (12/2010[a,b])	2(2)	2800/3600	n.e.	>130 (n.r.)	69±1	3(2)	–4.3/–2.8	–3.7±0.5	No clear change (Insufficient data)
			After (05/2016)	110(89)	3.4/5300	62.4±1.1	54±11	32±4	3(2)	–3.0/–2.0	–2.6±0.6	
	Alluvial Terrace	28.33718°,84.39898°,942m	After (05/2016, 01/2017, 01/2018)	386(309)	2.5/28,700	90.4±1.1	360±80	46±4	8(6)	–3.6/+0.0	–2.0±0.4	Unknown
	Hot Spring TATMARS4B	28.33707°,84.39903°,945m	After (05/2016)	0			n.e.	7.5±0.5	1(1)		–6.8±0.1	Unknown
	Southern Springs	28.32988°,84.40058°,934m	After (01/2017, 01/2018)	14(14)	7.7/150	28.5±2.9	n.e.	10±1	1(1)		–6.9±0.1	Unknown
**Budhi Gandaki Valley**												
Khorlabesi	Gas Zone	28.25268°,84.87913°,957m	After (01/2017, 01/2018)	32(25)	2.1/2600	41.5±1.6	>40±10	14±3	2(1)	–5.6/–5.4	–5.5±0.1	Unknown
Machhakhola	Near Hot Spring	28.23120°,84.87572°,804m	After (01/2017, 01/2018)	63(43)	21.6/81,800	514±16	>1240±330	91±2	4(4)	–3.7/–3.0	–3.2±0.2	Increase (New CO_2 emission)
**Upper Trisuli Valley**												
Sanjen	Tunnel	28.21783°,85.28425°,2187m	After (11/2016, 01/2018)	36(33)	13.2/94,700	640±40	>580±150	97±1	4(4)	–0.7/+0.1	–0.1±0.2	Increase (New CO_2 emission)
	Piezometer DH1	28.21765°,85.28392°,2183m	After (01/2016, 05/2016)	3(1)		92,900±5200	n.e.	99.0±0.2	3(1)	–0.95/–0.93	–0.94±0.01	Increase (New CO_2 emission)
	Piezometer DH2	28.21785°,85.28420°,2181m	After (01/2016, 05/2016)	3(1)		15,400±2900	n.e.	97.7±0.4	4(1)	–0.73/–0.67	–0.70±0.01	Increase (New CO_2 emission)
Chilime	Gas Zone	28.22095°,85.29860°,2648m	Before (08/2010[a,c], 01/2011[a,c])	192(92)	3.4/123,000	608±12	470±120	98±1	2(2)	–1.6/–1.3	–1.5±0.1	Decrease (Decreasing CO_2 emission)
			After (11/2015, 01/2016)	138(131)	2.6/10,100	136±2	91±19	n.m.	3(3)	–1.6/–1.4	–1.50±0.06	
			After (01/2018)	99(93)	0.3/2620	21.3±2.0	19±4	n.m.	0		n.m.	
Syabru-Bensi	GZ1–2 Terrace	28.16283°,85.33765°,1424m	Before (2006–2011[a,d,e])	652(333)	2.5/236,000	196±2	480±50	94±1	7(3)	–1.27/–0.77	–0.88±0.07	Increase (Higher CO_2 emission)
			After (2015–2018)	577(378)	2.8/226,000	236±3	1010±110	97±2	14(4)	–0.92/–0.53	–0.74±0.02	
	GZ3 Gas Zone	28.15925°,85.33513°,1410m	Before (2007–2011[a,d])	580(139)	2.0/19,000	299±6	740±130	80±1	1(1)		–0.5±0.1	No clear change (Insufficient data)
			After (11/2016, 09/2017)	5(1)		22,000±4500	n.e.	84±1	2(1)	–0.78/–0.73	–0.76±0.02	
	Hot Spring FF2	28.15998°,85.33675°,1394m	After (01/2016)	0			n.e.	n.m.	2(1)	–2.38/–1.97	–2.2±0.1	Increase (New CO_2 emission)
	Hot Spring SBE1	28.16362°,85.33972°,1415m	Before (01/2011[a])	0			n.e.	36±1	1(1)		–3.6±0.1	Unknown
North Syabru	Hot Spring TT1	28.18347°,85.34367°,1469m	Before (11/2003[b], 01/2011[a])	0			n.e.	98±1	2(1)	–2.2/–1.97	–2.1±0.1	No clear change (Insufficient data)
			After (01/2016)	0			n.e.	n.m.	1(1)		–2.0±0.1	
	Hot Spring MEH1	28.19582°,85.35060°,1493m	After (01/2016)	0			n.e.	n.m.	1(1)		–2.4±0.1	Unknown
Timure	All Gas Zones		Before (2008–2011[a,b,c])	294(243)	0.7/11,100	95.4±1.1	830±170	24±5	3(3)	–2.9/–0.6	–1.7±0.7	Unknown
	Northern Profile	28.24213°,85.35895°,1666m	Before (01/2010[c], 01/2011[c])	69(44)	73/5600	767±22	91±23	17.0±0.5	1(1)		–1.6±0.1	Increase (Increasing CO_2 emission)
			After (11/2015, 01/2016)	75(69)	110/17,400	1850±50	270±70	n.m.	2(2)	–2.12/–2.09	–2.11±0.02	
			After (09/2017)	117(89)	6.1/175,000	2170±50	740±200	n.m.	0		n.m.	

n.e.: not estimated
n.r.: not reliable
n.m.: not measured
N_{meas}(n_{point}): total number of measurements (total number of measurement points)
[a] Data from ref. 30
[b] Data from ref. 52
[c] Data from ref. 51
[d] Data from ref. 34
[e] Data from refs. 33 and 62

Supplementary Table 2 | Available data of temperature, pH, flow rate, and dissolved inorganic carbon concentration and isotopic ratio of hot springs before and after the Gorkha earthquake at eighteen hydrothermal sites in Central Nepal.

Site	Location	Name	Type	Coordinates	Before/After Gorkha Earthquake	Spring temperature (°C)	Spring pH	Spring flow rate (L s^{-1})	Dissolved Inorganic Carbon (DIC)			Post-seismic Effect?
									N_{meas}	C_{DIC} (mmol L^{-1})	$\delta^{13}C_{DIC}$ (‰) (V-PDB)	
**Marsyandi Valley**												
Khudi	Probi Hot Springs	PRO1	HS	28.34545°,84.33100°,1259m	After (01/2017)	35.6±0.1	6.6±0.1	n.m.	1	35.8±0.2	2.2±0.2	Unknown
		PRO2	HS,BB	28.34515°,84.33135°,1250m	After (01/2017)	30.1±0.1	6.3±0.1	n.m.	1	36.3±0.2	2.1±0.2	Unknown
		PRO3	HS	28.34547°,84.33112°,1250m	After (01/2017)	27.9±0.1	6.4±0.1	n.m.	1	32.5±0.2	1.9±0.2	Unknown
		PRO4	HS	28.34638°,84.33170°,1289m	After (01/2017)	24.1±0.1	6.2±0.1	1.0±0.3	1	8.1±0.3	–2.0±0.4	Unknown
Bahundanda	Main Hot Springs	TATMARS2	HS,DDS	28.33992°,84.39775°, 930m	Before (03/1999[b], 03/2001[b], 05/2002[a], 10/2003[a], 12/2010)	49.7±0.2	6.3±0.2	0.50±0.03	6	38.7±4.4	10.7±1.2	No change
					After (05/2016, 01/2017, 01/2018)	48.1±0.3	6.7±0.7	0.33±0.04	1	38.6±0.3	0.2±0.2	
		TATMARS3	HS	28.33955°,84.39695°,935m	Before (10/2003[a], 12/2010)	46.0±0.1	6.3±0.1	0.13±0.01	1	9.2±0.9	12.5±0.1	No change
					After (05/2016, 01/2017, 01/2018)	46.5±0.9	6.4±0.4	0.032±0.001	1	39.0±0.3	–0.2±0.3	
		TATMARS5	HS	28.34033°,84.39782°,957m	After (05/2016, 01/2017)	45.1±0.1	6.6±0.7	n.m.	1	44.8±0.4	0.1±0.2	Unknown
		TATMARS6	HS	28.34017°,84.39785°,952m	After (05/2016, 01/2017)	37.3±0.1	6.5±0.5	n.m.	1	37.3±0.2	0.1±0.2	Unknown
	Alluvial Terrace	TATMARS4A	HS,DDS	28.33718°,84.39898°,942m	Before (10/2003[a])	32.3±0.1	6.1±0.1	n.m.	1	12.6±1.3	11.9±0.1	No change
					After (05/2016, 01/2017)	34.8±0.1	6.3±0.3	n.m.	1	36.4±0.2	–0.9±0.2	
		TATMARS4B	HS,BB,DDS	28.33707°,84.39903°,945m	After (12/2010, 05/2016, 01/2017, 01/2018)	33.5±0.2	6.7±0.5	n.m.	1	41.9±0.4	–0.6±0.3	Unknown
		TATMARS4C	HS,DDS	28.33693°,84.39907°,947m	After (05/2016, 01/2017)	30.8±0.4	6.3±0.1	n.m.	1	42.3±0.3	–0.5±0.2	Unknown
		TATMARS4D	HS,DDS	28.33707°,84.39893°,947m	After (01/2017)	34.2±0.1	6.0±0.1	n.m.	1	40.1±0.2	–0.5±0.2	Unknown
	Southern Springs	TATMARS7A	HS	28.33450°,84.39855°,947m	After (01/2017)	32.7±0.1	6.1±0.1	n.m.	1	45.1±0.4	1.7±0.2	Unknown
		TATMARS7B	HS	28.33442°,84.39823°,959m	After (01/2017)	26.1±0.1	6.3±0.1	n.m.	1	41.4±0.3	2.0±0.2	Unknown
		TATMARS8A	HS	28.32978°,84.40040°,933m	After (01/2017, 01/2018)	22.9±0.1	6.2±0.1	n.m.	1	33.9±0.2	–2.0±0.3	Unknown
		TATMARS8B	HS,DDS	28.32988°,84.40058°,934m	After (01/2017, 01/2018)	23.1±0.1	6.3±0.1	n.m.	1	37.9±0.2	–2.1±0.2	Unknown
Shirchaur		SHIR1	HS	28.39830°,84.40740°,1067m	Before (04/2002[a], 10/2003[a])	31.1±0.1	7.9±0.1	n.m.	1	6.2±0.6	–3.8±0.1	No change
					After (11/2016)	36.9±0.1	8.1±0.1	0.115±0.002	1	7.9±0.1	–2.5±0.1	
Jagat	Southern Spring	JAG1	HS	28.41255°,84.40777°,1189m	Before (03/2001[b], 05/2002[a], 10/2003[a])	55.9±1.4	5.57±0.01	n.m.	4	10.9±1.1	9.9±0.8	No change
					After (11/2016)	53.3±0.1	5.3±0.1	1.21±0.04	1	7.0±0.1	–3.2±0.1	
	Northern Springs	JAG2A	HS	28.42145°,84.40400°,1202m	Before (03/1999[b], 03/2001[b], 04/2002[a])	52.3±1.2	7.75±0.04	n.m.	3	3.4±0.3	–9.2±0.9	No change
					After (11/2016)	52.0±0.1	7.6±0.1	n.m.	1	3.4±0.1	–3.6±0.1	
		JAG2B	HS	28.42167°,84.40375°,1237m	Before (10/2003[a])	42.3±0.1	7.9±0.1	n.m.	1	1.3±0.1	–13.8±0.1	No change
					After (11/2016)	46.9±0.1	7.8±0.1	n.m.	1	1.6±0.1	–8.7±0.1	
**Near Gorkha**												
Bhulbule		BHUL1	HS	28.01738°, 84.54955°,480m	Before (04/1995[b])	34.8±0.1	10.1±0.1	n.m.	1	75.8±7.6	–14.1±0.1	No change
					After (05/2016)	34.6±0.2	10.3±0.1	n.m.	0	n.m.	n.m.	
**Budhi Gandaki Valley**												
Machhakhola	Eastern Bank	BUD0	HS*,BB,DDS	28.23120°,84.87572°,804m	After (01/2017, 01/2018)	59.8±0.2	6.2±0.1	n.m.	1	26.4±0.3	–0.5±0.2	Increase (New)
		BUD0A	HS	28.23090°,84.87575°,814m	After (01/2018)	47.7±0.1	7.2±0.1	n.m.	0		n.m.	Unknown
		BUD0B	HS	28.23085°,84.87572°,820m	After (01/2018)	38.8±0.1	7.6±0.1	n.m.	0		n.m.	Unknown
		BUD0C	HS	28.23082°,84.87585°,812m	After (01/2018)	56.0±0.1	6.5±0.1	n.m.	0		n.m.	Unknown
		BUD0D	HS	28.23078°,84.87582°,818m	After (01/2018)	55.3±0.1	7.2±0.1	n.m.	0		n.m.	Unknown
		BUD0E	HS	28.23072°,84.87573°,844m	After (01/2018)	51.3±0.1	7.3±0.1	n.m.	0		n.m.	Unknown
	Western Bank	BUD6	HS	28.23365°,84.87582°,805m	After (01/2018)	57.1±0.1	6.6±0.1	n.m.	1	23.1±0.5	0.9±0.1	Unknown
Khorlabesi	Southern Spring	BUD1	HS,DDS	28.25268°,84.87913°,957m	After (01/2017)	31.6±0.1	6.4±0.1	0.6±0.2	0	n.m.	n.m.	Unknown
	Northern Spring	BUD2	HS	28.26612°,84.89080°,857m	Before (10/1999[b])	52±1	6.0±0.1	n.m.	1	7.6±0.8	n.m.	No change
					After (01/2017, 01/2018)	50±2	5.7±0.1	n.m.	1	14.4±0.4	–0.1±0.1	
Lisyapu		BUDX1	HS	28.27050°,84.89440°,900m	After (01/2018)	25.1±0.1	6.2±0.1	n.m.	1	21.3±0.2	0.7±0.1	Unknown

Tatopani	Southern Secondary Springs	BUD3B	HS*	28.27485°,84.89860°,929m	After (01/2017)	43.2±0.1	6.4±0.1	n.m.	1	25.6±0.9	0.9±0.3	**Increase (New)**
	Main Springs	BUD4B	HS	28.27542°,84.89857°,931m	Before (10/1999[b])	30±1	7.0±0.1	n.m.	1	12.2±1.2	n.m.	**Decrease (Temp.)**
					After (01/2017, 01/2018)	22.2±1.8	6.9±0.1	0.061±0.002	1	15.8±0.3	3.7±0.3	
		BUD4C	HS	28.27542°,84.89857°,931m	Before (10/1999[b])	50±1	6.9±0.1	n.m.	1	19.1±1.9	3.3±0.3	**Decrease (Temp.)**
					After (01/2017, 01/2018)	48.5±1.3	8.6±0.1	0.19±0.04	1	22.0±0.2	1.6±0.3	
	Northern Secondary Springs	BUD5A	HS	28.27573°,84.89863°,908m	After (01/2018)	40.0±0.1	8.2±0.1	n.m.	1	16.8±0.6	3.0±0.1	**Unknown**
		BUD5B	HS	28.27600°,84.89858°,905m	After (01/2018)	30.5±0.1	6.5±0.1	n.m.	1	14.3±0.3	1.7±0.1	**Unknown**
**Upper Trisuli Valley**												
Sanjen	Tunnel	TSJ3	HS*,DDS	28.21783°,85.28425°,2187m	After (11/2016)	20.3±0.1	6.6±0.1	n.m.	1	>55	–9.1±0.7	**Increase (New)**
	Piezometer	DH1	AQ,DDS	28.21765°,85.28392°,2183m	After (01/2016, 05/2016)	18.9±0.6	6.06±0.03	n.m.	2	43.8±0.1	1.6±0.1	**Unknown**
	Piezometer	DH2	AQ,DDS	28.21785°,85.28420°,2181m	After (01/2016, 05/2016)	19.1±0.8	5.95±0.07	n.m.	1	43.3±0.1	2.1±0.1	**Unknown**
Brapche		BRA1D	HS	28.20210°,85.28995°,1893m	After (01/2016, 11/2016)	43.8±0.1	6.7±0.1	n.m.	2	7.8±1.4	–4.4±1.2	**Unknown**
Chilime	Hot Spring	CHI	HS,DDS	28.22028°, 85.29778°,2648m	Before (10/1975[c], 03/1980[d], 04/1995[b], 11/2003[a], 01/2004[f], 01/2011[e], 04/2015)	48.9±0.4	6.1±0.3	5.0±0.2	3	13.8±1.3	8.3±0.4	**Decrease (Cessation)**
					After (11/2015, 01/2016, 01/2018)	no spring		0	0			
	Other Springs	CC1	HS	28.22125°,85.29903°,2670m	After (01/2016, 01/2018)	23.0±0.7	6.8±0.1	n.m.	2	3.0±0.1	–7.5±0.3	**Unknown**
		CC2	HS	28.22153°,85.29842°,2668m	After (01/2016, 01/2018)	19.8±0.1	6.4±0.1	n.m.	1	2.4±0.6	–9.8±0.6	**Unknown**
Bharku		BAR	HS	28.13253°,85.30222°,1347m	After (01/2016)	49.9±0.1	7.7±0.2	n.m.	1	4.5±0.5	–7.7±0.3	**Unknown**
Syabru-Bensi	Western Bank Secondary Springs	GZ3	HS,DDS	28.15908°,85.33556°,1422m	Before (12/2008, 01/2011)	20.3±1.2	6.6±0.1	n.m.	1	1.9±0.1	n.m.	**Increase (Temp.)**
					After (01/2016, 09/2017)	23.4±0.1	6.0±0.5	n.m.	1	3.2±0.1	3.7±0.1	
		FF1	HS	28.15993°,85.33633°,1410m	After (01/2016, 09/2017)	34.2±0.1	6.1±0.1	0.125±0.001	1	28.6±0.1	1.6±0.1	**Unknown**
		FF2	HS*,BB	28.15998°,85.33675°,1394m	After (01/2016)	in Trisuli River	n.m.	n.m.	0	n.m.	n.m.	**Increase (New)**
	Main Springs	SBP0	HS,DDS	28.16272°,85.33775°,1407m	Before (12/1980[d], 02/2001[b], 11/2003[a], 01/2004[f], 08/2007[f], 09/2007[f], 12/2007, 12/2008, 01/2011, 04/2015)	60.7±0.1	6.63±0.09	0.087±0.004	4	25.8±1.6	4.7±0.7	**Increase (Temp., flow, C_{DIC}, $\delta^{13}C_{DIC}$)**
					After (11/2015, 01/2016, 05/2016, 11/2016, 03/2017, 05/2017, 09/2017, 01/2018)	64.1±0.3	6.36±0.08	0.104±0.007	5	34.3±1.5	1.0±0.1	
		SBB5	HS,DDS	28.16272°,85.33775°,1407m	Before (08/2007[f], 09/2007[f], 12/2007, 12/2008, 01/2011)	31.8±0.3	6.03±0.09	0.282±0.009	2	25.8±3.6	0.9±0.1	**Increase (Temp., flow)**
					After (11/2015, 01/2016, 11/2016, 05/2017, 09/2017, 01/2018)	34.9±0.2	6.0±0.1	0.37±0.02	4	29.6±0.9	0.2±0.3	
		SBC1	HS,DDS	28.16272°,85.33775°,1407m	Before (12/2007, 01/2011)	35.55±0.04	6.5±0.1	n.m.	1	15.1±0.3	2.7±0.1	**No change**
					After (01/2016, 09/2017, 09/2017–01/2018)	32.6±0.8	6.53±0.06	n.m.	1	17.7±0.1	n.m.	
		SBC2	HS,DDS	28.16272°,85.33775°,1407m	Before (01/2004[f], 12/2007, 12/2008, 01/2011)	50.1±1.9	6.8±0.2	n.m.	2	17.8±1.1	2.6±0.1	**Decrease (Temp.)**
					After (11/2015, 01/2016)	40.2±0.5	6.47±0.02	n.m.	1	22.3±0.1	2.1±0.1	
		SBM	HS*,DDS	28.16272°,85.33775°,1407m	After (11/2015, 01/2016, 09/2017, 09/2017–01/2018)	34.9±0.2	6.03±0.04	n.m.	1	30.3±0.1	–0.6±0.1	**Increase (New)**
		SBN	HS*,DDS	28.16272°,85.33775°,1407m	After (11/2015, 01/2016, 05/2016)	37.3±0.8	6.4±0.1	n.m.	1	27.4±0.1	2.3±0.1	**Increase (New)**
		SBN2	HS*,DDS	28.16272°,85.33775°,1407m	After (05/2016, 11/2016, 09/2017, 09/2017–01/2018)	39.2±0.6	6.53±0.06	0.50±0.02	3	17.3±1.1	2.0±0.2	**Increase (New)**
	Eastern Bank Secondary Springs	SBE1	HS,BB	28.16362°,85.33972°,1415m	Before (12/2007, 01/2011)	53.6±1.3	6.6±0.2	n.m.	1	17.4±0.5	0.5±0.1	**No clear change**
					After (01/2016)	50.2±0.1	6.4±0.1	n.m.	1	26.4±0.1	1.1±0.1	
		SBE2	HS	28.16362°,85.33972°,1415m	Before (12/2007, 01/2011)	46.1±1.8	6.9±0.1	n.m.	1	15.9±0.5	0.5±0.1	**No clear change**
					After (01/2016)	40.5±0.1	6.6±0.1	n.m.	1	19.4±0.1	1.6±0.1	
		PAS	HS,BB	28.16577°,85.34275°,1419m	Before (12/2008)	31.9±0.1	6.0±0.1	n.m.	1	42.9±1.3	n.m.	**No clear change**
					After (01/2016)	30.3±1.0	7.8±0.2	n.m.	1	32.9±0.1	–0.8±0.1	
		TS1	HS	28.16112°,85.33780°,1456m	Before (01/2007, 12/2007, 12/2008, 01/2011)	48.2±0.1	7.5±0.1	n.m.	2	18.8±0.6	2.6±0.1	**No change**
					After (01/2016)	47.1±0.1	7.0±0.1	n.m.	1	22.6±0.1	2.1±0.1	
		TS2	HS	28.16112°,85.33780°,1456m	After (01/2016)	26.9±0.1	8.0±0.1	n.m.	1	19.5±0.1	3.3±0.1	**Unknown**
		TS3	HS	28.16112°,85.33780°,1456m	After (01/2016)	47.9±0.1	7.9±0.1	n.m.	1	18.9±0.1	3.0±0.1	**Unknown**
		TS4	HS	28.16278°,85.33848°,1406m	After (01/2016)	37.2±1.0	7.6±0.2	n.m.	1	14.1±0.2	6.0±0.2	**Unknown**
		TSFG	HS	28.16302°,85.33905°,1406m	After (01/2016)	29.7±1.0	7.7±0.2	n.m.	1	26.1±0.1	2.5±0.1	**Unknown**
		TSFG2	HS	28.16075°,85.33773°,1406m	After (09/2017)	44.2±0.1	7.6±0.2	n.m.	0	n.m.	n.m.	**Unknown**

North Syabru	Western Bank	TT1	HS,BB	28.18347°,85.34367°,1469m	Before (01/2001[b], 11/2003[a], 01/2011)	24.38±0.07	5.61±0.05	n.m.	4	35.8±2.9	12.3±0.7	**Increase (Temp.)**
					After (11/2015, 01/2016, 01/2018)	25.3±0.1	5.3±0.1	n.m.	2	38.3±2.3	–0.6±0.1	
	Eastern Bank	MEH1	HS,BB	28.19582°,85.35060°,1493m	After (01/2016)	60.5±0.1	6.48±0.02	n.m.	1	30.9±0.1	0.9±0.1	**Unknown**
Timure		TIM	HS,DDS	28.24092°,85.35867°,1676m	Before (04/1995[b], 11/2003[a], 12/2008[e], 01/2011[e], 04/2015)	61.3±2.2	6.5±0.1	0.20±0.02	5	17.3±1.0	3.3±0.1	**Increase (Temp.)**
					After (11/2015, 01/2016, 09/2017, 11/2017)	70.7±1.0	6.3±0.1	n.m.	2	14.2±0.2	2.1±0.1	
Langtang		LPAH	HS	28.15164°,85.37250°,1666m	Before (01/2001[a], 01/2011[d], 11/2014)	41.0±0.1	6.4±0.3	n.m.	2	5.1±0.2	–4.8±0.1	**Decrease (Temp.)**
					After (01/2016)	37.3±0.5	n.m.	n.m.	1	4.8±0.2	–3.2±0.2	
**Bhote Koshi Valley**												
Kodari		KOD	HS	27.94695°,85.95160°,1493m	Before (03/1995[b], 01/2000[f], 01/2011)	44.6±1.2	6.6±0.1	3.0±0.2	1	8.4±0.3	–8.7±0.1	**Increase (Temp.)**
					After (05/2016)	50.7±0.1	7.2±0.1	3.00±0.04	0	n.m.	n.m.	
		KOD2	HS*	27.94692°,85.95152°,1500m	After (05/2016)	48.1±0.1	6.5±0.1	n.m.	0	n.m.	n.m.	**Increase (New)**

n.m.: not measured

* new spring that appeared after the Gorkha earthquake

HS: hot spring; BB: bubbles; DDS: diffuse degassing structure; AQ: aquifer degassing

Compilation of new original data and of data compiled in ref. 30, and in particular from: [a] refs. 31 and 52; [b] ref. 32; [c] ref. 63; [d] ref. 64; [e] ref. 51; [f] ref. 33.

Supplementary Table 3 | Vertical Peak Ground Velocity, Seismic Energy Density and Peak Dynamic Stress estimated for Gorkha earthquake and six of the main aftershocks, at eight hydrothermal sites, and three specific seismic events near Syabru-Bensi. (see Methods)

**Location**	**Marsyandi Valley**	**Budhi Gandaki Valley**		**Upper Trisuli Valley**				**Bhote Koshi Valley**
**Site**	**Bahundanda**	**Machhakhola**	**Tatopani**	**Sanjen**	**Chilime**	**Syabru-Bensi**	**Timure**	**Kodari**
**M_w7.8 Gorkha earthquake / April 25, 2015 11:56 (local time) / 28.115°, 84.772°, depth: 14.5 km**								
Epicentral distance (km)	44.7	16.5	21.8	51.9	53.4	56.2	59.7	118.3
Peak Ground Velocity (cm s^{-1})	31.2	61.6	51.4	28.0	27.4	26.5	25.3	15.5
Peak Dynamic Stress (MPa)	2.7	5.3	4.4	2.4	2.4	2.3	2.2	1.3
Seismic Energy Density (J m^{-3})	130	2600	1100	80	74	63	53	6.6
**M_L6.0 aftershock / April 25, 2015 12:00 (local time) / 28.420°, 84.934°, depth: 12 km**								
Epicentral distance (km)	53.8	21.7	16.4	41.3	42.3	49.1	46.5	113.6
Peak Ground Velocity (cm s^{-1})	3.8	10.0	13.3	5.0	4.9	4.2	4.4	1.6
Peak Dynamic Stress (MPa)	0.32	0.86	1.1	0.43	0.42	0.36	0.38	0.14
Seismic Energy Density (J m^{-3})	0.17	2.7	6.3	0.39	0.36	0.23	0.27	0.02
**M_L5.8 aftershock / April 25, 2015 12:05 (local time) / 28.053°, 85.001°, depth: 12 km**								
Epicentral distance (km)	67.7	23.4	26.7	33.4	34.8	35.5	41.1	94.8
Peak Ground Velocity (cm s^{-1})	2.1	7.2	6.2	4.8	4.6	4.5	3.8	1.4
Peak Dynamic Stress (MPa)	0.18	0.62	0.53	0.41	0.39	0.39	0.33	0.12
Seismic Energy Density (J m^{-3})	0.04	1.1	0.74	0.38	0.33	0.31	0.10	0.02
**M_L6.7 aftershock / April 25, 2015 12:30 (local time) / 28.221°, 84.909°, depth: 12 km**								
Epicentral distance (km)	52.3	3.5	6.1	37.1	38.6	42.9	44.6	107.6
Peak Ground Velocity (cm s^{-1})	9.6	65.8	54.4	13.4	12.9	11.6	11.2	4.8
Peak Dynamic Stress (MPa)	0.83	5.6	4.7	1.1	1.1	1.0	0.96	0.41
Seismic Energy Density (J m^{-3})	2.0	7300	1300	5.6	5.0	3.6	3.2	0.22
**M_L6.7 aftershock / April 26, 2015 12:54 (local time) / 27.737°, 86.039°, depth: 15.6 km**								
Epicentral distance (km)	175.7	127.6	127.8	91.8	90.9	84.0	87.6	24.9
Peak Ground Velocity (cm s^{-1})	3.0	4.1	4.1	5.6	5.7	6.1	5.9	19.4
Peak Dynamic Stress (MPa)	0.26	0.35	0.35	0.48	0.49	0.52	0.50	1.7
Seismic Energy Density (J m^{-3})	0.05	0.13	0.13	0.36	0.37	0.47	0.41	18.8
**M_L7.3 aftershock / May 12, 2015 12:50 (local time) / 27.793°, 86.172°, depth: 18.5 km**								
Epicentral distance (km)	185.9	137.2	137.0	99.8	98.6	92.3	94.6	27.7
Peak Ground Velocity (cm s^{-1})	6.3	8.2	8.2	10.6	10.7	11.4	11.1	30.5
Peak Dynamic Stress (MPa)	0.54	0.70	0.70	0.91	0.92	0.97	0.95	2.6
Seismic Energy Density (J m^{-3})	0.32	0.79	0.80	2.1	2.2	2.6	2.4	101
**M_L6.3 aftershock / May 12, 2015 13:21 (local time) / 27.659°, 86.217°, depth: 6.9 km**								
Epicentral distance (km)	195.3	147.2	147.4	111.3	110.3	103.5	106.7	41.4
Peak Ground Velocity (cm s^{-1})	1.5	2.0	2.0	2.6	2.7	2.9	2.8	7.5
Peak Dynamic Stress (MPa)	0.12	0.17	0.17	0.23	0.23	0.24	0.24	0.64
Seismic Energy Density (J m^{-3})	0.01	0.02	0.02	0.05	0.05	0.07	0.06	1.1
**M_L5.4 aftershock / August 28, 2015 06:03 (local time) / 28.005°, 85.295°, depth: 2 km**								
Epicentral distance (km)				23.6	23.9	18.0	26.9	
Peak Ground Velocity (cm s^{-1})				4.3	4.2	5.8	3.7	
Peak Dynamic Stress (MPa)				0.37	0.36	0.50	0.31	
Seismic Energy Density (J m^{-3})				0.32	0.31	0.73	0.21	
**M_L4.4 aftershock / October 31, 2015 03:43 (local time) / 28.041°, 85.237°, depth: 2 km**								
Epicentral distance (km)				20.2	20.9	16.8	25.2	
Peak Ground Velocity (cm s^{-1})				0.89	0.85	1.1	0.65	
Peak Dynamic Stress (MPa)				0.076	0.073	0.097	0.056	
Seismic Energy Density (J m^{-3})				0.015	0.013	0.025	0.0073	
**M_L4.6 aftershock / November 17, 2015 13:10 (local time) / 28.005°, 85.271°, depth: 10 km**								
Epicentral distance (km)				23.6	24.1	18.7	27.6	
Peak Ground Velocity (cm s^{-1})				1.1	1.1	1.6	0.93	
Peak Dynamic Stress (MPa)				0.098	0.096	0.13	0.080	
Seismic Energy Density (J m^{-3})				0.021	0.020	0.043	0.013	
