# Supplementary material for: Persistent CO2 emissions and hydrothermal unrest following the 2015 earthquake in Nepal
Source: Nat Commun. 2018 Jul 27;9:2956. doi: 10.1038/s41467-018-05138-z (PMC6063904; doi:10.1038/s41467-018-05138-z)
Supplement: Supplementary file 3 — Description of Additional Supplementary Files [file 41467_2018_5138_MOESM3_ESM.pdf]

Description of Additional Supplementary Files

File Name: Supplementary Movie 1
Description: Significant carbon dioxide emission from the ground of a tunnel in Sanjen, Upper Trisuli valley (video recorded on November 24th, 2016). In January 2018, the CO2 is still emitted, and about 4 to 5 vol% of CO2 is measured in the air of the tunnel.

File Name: Supplementary Movie 2
Description: Large carbon dioxide degassing from the Machhakhola hot spring, Budhi Gandaki valley (video recorded on January 25th, 2017). This hot spring and the CO2 emission occurred for the first time after the Gorkha earthquake. They still exist in January 2018. The CO2 flux through the water layer was measured at the strongest bubbling location (see Methods and Supplementary Fig. 8).
